# Unmasking the hidden risk: potential implication of pullback pressure gradient in ischemia-negative lesions

**DOI:** 10.1007/s12928-025-01147-0

**Published:** 2025-07-02

**Authors:** Hirohiko Ando, Carlos Collet, Koshiro Sakai, Hirofumi Ohashi, Tetsuya Amano

**Affiliations:** 1 Department of Cardiology, Aichi Medical University, 1-1 Yazakokarimata, Nagakute, Japan; 2 Cardiovascular Center Aalst, OLV Clinic, Aalst, Belgium

Percutaneous coronary intervention is generally not recommended for lesions without evidence of ischemia due to limited clinical benefit. However, acute coronary syndrome can arise from ischemia-negative lesions. Identifying reliable predictors for plaque rupture in such cases remains challenging. Recently, a new concept combining plaque morphology and hemodynamic factors has been proposed to better stratify high-risk non-ischemic lesions.

A 68-year-old man presented with atypical chest symptoms. Coronary angiography revealed significant stenosis in the left circumflex artery. Physiological assessment showed a resting full-cycle ratio of 0.98 and a fractional flow reserve of 0.92, indicating no ischemia. However, pullback tracing demonstrated a high pullback pressure gradient (PPG) of 0.92, suggesting a focal pattern of disease. In the absence of ischemia, revascularization was deferred, and the patient was managed medically (Fig. 1).

**Fig. 1:**
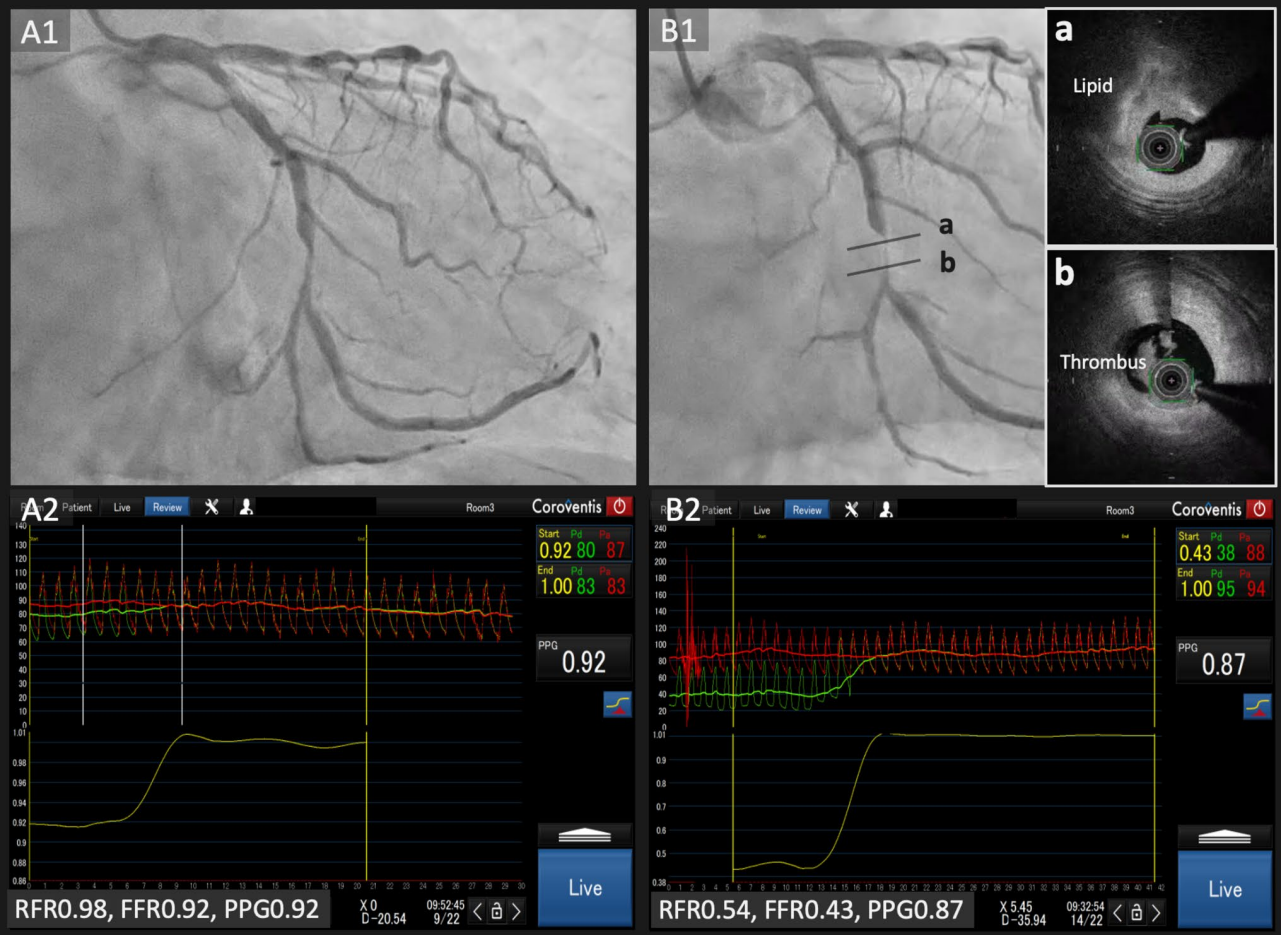
**A1** Baseline angiography showing significant stenosis in the left circumflex artery. **A2** Physiological assessment demonstrated no ischemia, but a high pullback pressure gradient was observed. **B1** Emergent angiography performed after non-ST-elevation myocardial infarction, showing progression of the lesion. Optical frequency-domain imaging revealed a lipid-rich plaque with thrombus, indicating a recent plaque rupture. **B2** Physiological assessment during the emergent procedure demonstrating worsened pressure indices. *RFR* resting full-cycle ratio, *FFR* fractional flow reserve, *PPG* pullback pressure gradient

One year later, the patient developed non-ST-elevation myocardial infarction. Emergent angiography demonstrated progression of the lesion, with further reduction in physiological indices. Intravascular imaging revealed a vulnerable plaque with thrombus formation, which was treated successfully with drug-eluting stent implantation (Supplementary Movie).

PPG is a novel physiological index that characterizes coronary artery disease as either focal or diffuse, based on hyperemic pressure pullback curves. Values approaching 0 indicate diffuse disease, whereas values close to 1 represent focal patterns [1, 2]. This case highlights that a high PPG, reflecting a steep pressure drop across a lesion, may predict future adverse events even in the absence of ischemia. Previous studies have demonstrated a strong association between elevated PPG values and plaque vulnerability [3]. It is further hypothesized that such a translesional pressure gradient imposes mechanical stress on the plaque, potentially contributing to rupture. Therefore, incorporating PPG into standard physiological assessment may enhance risk stratification and inform decisions regarding revascularization.

Assessing PPG may provide additional insight into plaque stability and support more tailored management strategies. Further studies are needed to validate the clinical utility of PPG as a predictive marker.

## Supplementary Information

Below is the link to the electronic supplementary material.

Supplementary file1 (MP4 1277 KB)
