# Correction: Unmasking the hidden risk: potential implication of pullback pressure gradient in ischemia-negative lesions

**DOI:** 10.1007/s12928-025-01201-x

**Published:** 2025-09-30

**Authors:** Hirohiko Ando, Carlos Collet, Koshiro Sakai, Hirofumi Ohashi, Tetsuya Amano

**Affiliations:** 1 Department of Cardiology, Aichi Medical University, 1-1 Yazakokarimata, Nagakute, Japan; 2 Cardiovascular Center Aalst, OLV Clinic, Aalst, Belgium

**Correction: Cardiovascular Intervention and Therapeutics (2025) 40:1007–1008** 10.1007/s12928-025-01147-0

The article "Unmasking the hidden risk: potential implication of pullback pressure gradient in ischemia‑negative lesions", written by Hirohiko Ando, Carlos Collet, Koshiro Sakai, Hirofumi Ohashi and Tetsuya Amano, was originally published under exclusive license to The Author(s) under exclusive licence to Japanese Association of Cardiovascular Intervention and Therapeutics. As a result of the subsequent decision to publish the article under the open access model, the article’s copyright notice was changed on 17 September 2025 to © The Author(s) 2025 and the article is now distributed under a Creative Commons Attribution 4.0 International License, which permits use, sharing, adaptation, distribution and reproduction in any medium or format, as long as you give appropriate credit to the original author(s) and the source, provide a link to the Creative Commons licence, and indicate if changes were made. The images or other third party material in this article are included in the article's Creative Commons licence, unless indicated otherwise in a credit line to the material. If material is not included in the article's Creative Commons licence and your intended use is not permitted by statutory regulation or exceeds the permitted use, you will need to obtain permission directly from the copyright holder. To view a copy of this licence, visit http://creativecommons.org/licenses/by/4.0

The original article has been corrected.

